# Antimicrobial gelatin-based films with cinnamaldehyde and ZnO nanoparticles for sustainable food packaging

**DOI:** 10.1038/s41598-024-72009-7

**Published:** 2024-09-28

**Authors:** Maha Sultan, Hassan Ibrahim, Hossam Mohammed El-Masry, Youssef R. Hassan

**Affiliations:** 1 Packaging Materials Department, National Research Centre, 33 El-Behouth St., P.O.12622, Dokki, Cairo Egypt; 2 Pre-Treatment and Finishing of Cellulosic Fibres Department, National Research Centre, 33 El-Behouth St., P.O.12622, Dokki, Cairo Egypt; 3 Chemistry of Natural and Microbial Products, National Research Centre, 33 El-Behouth St., P.O.12622, Dokki, Cairo Egypt

**Keywords:** Cinnamaldehyde, ZnO nanoparticles, Sorption isotherm, Barrier properties, Antioxidant capacity, Antimicrobial activity, Nanoscale materials, Structural materials

## Abstract

Cinnamaldehyde (CIN), a harmless bioactive chemical, is used in bio-based packaging films for its antibacterial and antioxidant properties. However, high amounts can change food flavor and odor. Thus, ZnO nanoparticles (NPs) as a supplementary antimicrobial agent are added to gelatin film with CIN. The CIN/ZnO interactions are the main topic of this investigation. FTIR-Attenuated Total Reflection (ATR), X-ray diffraction (XRD), and scanning electron microscopy (SEM) were utilized to investigate CIN/ZnO@gelatin films. Transmission electron microscope (TEM) images revealed nanospheres morphology of ZnO NPs, with particle sizes ranging from 12 to 22 nm. ZnO NPs integration increased the overall activation energy of CIN/ZnO@gelatin by 11.94%. The incorporation of ZnO NPs into the CIN@gelatin film significantly reduced water vapour permeability (WVP) of the CIN/ZnO@gelatin film by 12.07% and the oxygen permeability (OP) by 86.86%. The water sorption isotherms of CIN/ZnO@gelatin were described using Guggenheim-Anderson-de Boer (GAB) model. The incorporation of ZnO NPs into the CIN@gelatin film reduced monolayer moisture content (*M*_0_) by 35.79% and significantly decreased the solubility of CIN/ZnO@gelatin by 15.15%. The inclusion of ZnO into CIN@gelatin film significantly decreased tensile strength of CIN/ZnO@gelatin by 13.32% and Young`s modulus by 18.33% and enhanced elongation at break by 11.27%. The incorporation of ZnO NPs into the CIN@gelatin film caused a significant decrease of antioxidant activity of CIN/ZnO@gelatin film by 9.09%. The most susceptible organisms to the CIN/ZnO@gelatin film included *Candida albicans*, *Helicobacter pylori*, and *Micrococcus leutus*. The inhibition zone produced by the CIN/ZnO@gelatin film versus *Micrococcus leutus* was 25.0 mm, which was comparable to the inhibition zone created by antibacterial gentamicin (23.33 mm) and cell viability assessment revealed that ZnO/CIN@gelatin (96.8 ± 0.1%) showed great performance as potent biocompatible active packaging material.

## Introduction

Biodegradable polymers such as starch, collagen, gelatin, chitosan, polylactic acid, and alginate commonly contribute in producing packaging films as sustainable and renewable resources^1^. The food industry and consumers are very interested in antimicrobial biodegradable packaging materials not only as a way to improve food preservation and shelf life extension but also as a way to minimize the environmental issues caused by synthetic plastic packaging. A new concept referred to as antimicrobial packaging technology is a type of active packaging in which the environment, the product, and the package interact to prolong the period of lag and/or slow the growth rate of germs. As renewable and sustainable resources, biopolymers such as alginate, starch, collagen, Gelatin, and chitosan, are increasingly used in the production of packaging films^2–4^. With the intention of using biopolymers as bioactive coating for food packaging, for example, zein fibers loaded with phenolic-enriched extracts from the pulp, seed, and skin of orange chilto were assembled on polyhydroxyalkanoate (PHA) using the electrospinning technique^5^. Food products that are both lipophilic and hydrophilic can be preserved with the use of antioxidant coatings in food packaging structures. Covalent interaction between dialdehyde kappa-carrageenan (DAK-car) and zein nanoparticle content loaded with thymol was used to generate gelatin-based films. Thymol had satisfactory antioxidant and antibacterial properties at doses of 0.25 and 0.5 mg/mL^6^.

Nevertheless, these biopolymers have poor antioxidant capacities and are susceptible to microbial attacks. When antimicrobial packaging materials are used, the product’s shelf life is extended, and its quality and safety are better maintained. In order to improve the product’s health benefits while avoiding the toxicity of conventional active ingredients, natural antioxidant and non-toxic antimicrobials are also necessary^7–10^. Therefore, a modern alternative for food preservation and shelf life extension is the development of biodegradable antimicrobial polymers for food packaging applications, using natural antioxidant and antibacterial chemicals, such as essential oils or their active components and nanoparticles. Inorganic materials like calcium oxide (CaO), magnesium oxide (MgO), titanium oxide (TiO_2_), and zin oxide (ZnO) have attracted a lot of research attention recently with the development of nanotechnology because of their stability, resistance to harsh processing conditions, and good activity behavior against food-borne pathogens even though some of them are commercially available. Zinc oxide nanoparticles (ZnO NPs) are an attractive antibacterial agent because of their potent antibacterial action, non-toxicity, and considerable stability^11,12^. ZnO NPs has been used widely as a functional filler in UV absorbers for use in cosmetics, medicinal materials, pigments, and coating materials^13^.

It is essential to avoid food spoiling caused by bacteria and fungi to reduce health risks and avoid significant financial losses. However, there are still issues with traditional antibacterial and antifungal drugs that have persisted over time. These issues include drug interactions, toxicity of the chemical antimicrobial and antifungal components, drug resistance, and high costs^14^. Because of the toxicity and drug resistance of the currently available drugs, research into novel antimicrobial and antifungal agents is desperately needed. As a result, more researchers concern novel natural compounds derived from medicinal plants, like geraniol and cinnamaldehyde with the goal of resolving the issue of bacterial and fungal drug resistance while taking into account the products' inherent low toxicity and strong antimicrobial action^15^.

Cinnamaldehyde (CIN) is an unsaturated aldehyde and a phenolic terpenoid found in abundance in cinnamon, it is frequently utilized as a food ingredient. It has significant antioxidant, antifungal, antibacterial, and anti-inflammatory properties^16,17^.

As a natural antibacterial ingredient, CIN is widely used in the cosmetic, food, and pharmaceutical industries^16,18–20^. Additionally, it has been claimed that CIN inhibits the enzymes β -(1,3) glucan synthase and quitin synthase 1, both of which are crucial for the production of enzymes in the cell walls of yeasts and moulds and the interior membranes of various bacteria, including *E. coli* and *Staphylococcus aureus*^21^.

Numerous investigations have shown that CIN can be used as a possible antibacterial agent in a variety of concentrations. A biodegradable packaging film made of liquefied ball-milled shrimp shell chitin/polyvinyl alcohol (LBSC/PVA) blend films were developed. It contained inclusions of cyclodextrin/cinnamaldehyde (CD/CIN). The prolonged release of CIN was significantly improved by the host- guest interactions, which efficiently enclosed CIN in the cavity of CD. The performance of the blend films in terms of food preservation and antibacterial activity were considerably improved by the addition of the -CD/CIN inclusion. In addition, the 3 wt% -CD/CIN/LBSC/PVA blend film clearly exhibited more prolonged antibacterial activity and better cherry tomato preservation performance than the 3-wt% CIN/LBSC/PVA blend film, highlighting the crucial function of -CD in postponing the CIN release^22^.

CIN-loaded chitosan nanoparticles (CCNPs) were synthesized and inserted into chitosan/poly (vinyl alcohol)/fish gelatin (CPF) ternary matrices. According to release experiments, the CPF-CCNPs bionanocomposite film demonstrated CIN's sustained release behavior. Similarly, food-borne pathogens like Gram-positive (*Staphylococcus aureus* and *Listeria monocytogenes)* and Gram-negative (*Escherichia coli* and *Salmonella enteritidis*) bacteria were inhibited by the bioactive nanocomposites. At the highest CIN loading concentration, the bionanocomposite films demonstrated ferric reducing power and DPPH radical scavenging activity (∼ 16.4%) in vitro. Furthermore, the shelf life of rainbow trout fillets wrapped in CPF-CCNPs has extended to 12-day^23^.

Wu et al. reported that CIN and its sulphobutyl ether-β-cyclodextrin inclusion complex (CIN/S) were used to fabricate fish gelatin antibacterial composite films. The inclusion improved the films' ability to elongate at break and act as a light barrier. The best inhibitory ratio against *Pseudomonas aeruginosa* was shown by the film-forming solution combined with CIN and CIN/S, which was 98.43 (1.11%) in the first period and still 82.97 (4.55%) at 72 h. Additionally, the gelatin packaging solution used CIN and CIN/S to successfully stop microbial development while preserving the grass carp slices. The fact that the total volatile salt-based nitrogen (TVB-N) at the end of storage did not surpass 10 mg/100 g showed that the active coating undoubtedly increased the shelf life of the fish muscle^24^. Wang et al. developed chitosan /corn starch/ CIN film to preserve strawberries. The film's tensile strength was found to be as high as 31.24 ± 0.22 MPa at mass ratios of 2.5%, 7%, and 0.5% for chitosan, corn starch, and glycerin, respectively. The films' significant inhibitory impact on *Rhizopus*, *Escherichia coli*, and *Botrytis cinerea* was enhanced by the addition of CIN. Specifically, the film significantly improved the freshness of strawberries, minimizing the loss of nutrients while focusing on other strawberry metrics such as soluble solids, titratable acid value, and weight loss rate. As a result, the films can prolong the shelf life of strawberries to 11 days while also slowing down their physiological changes^25^.

Guo et al. have fabricated ZnO NPs/CIN/carboxymethylcellulose (CMC)-based packaging film. In turn, the CMC-based composite film including ZnO NPs and CIN showed good mechanical characterizations, significant water and oxygen molecule barriers, and anti-*Aspergillus niger* activities. Additionally, the ZnO/CIN/CMC nanocomposite film demonstrated a considerable reduction in the overall acidity content of cherry tomatoes during storage, as well as an inhibition of weight loss and firmness. Cherry tomatoes' quality can be enhanced by ZnO/CIN/CMC nanocomposite film packaging, which inhibits the fruits' physiological and metabolic processes during the postharvest storage phase^26^. High amylose corn starch-cinnamaldehyde (HACS-CIN) inclusion complex antibacterial films were reported by Wan et al. The release rate tests indicated that HACS-CIN inclusion films could reduce the rate of CIN volatilization, which demonstrated an encapsulation efficiency of up to 39.19%. With a tensile strength of 14.77 MPa and an elongation at break of 44.95%, the films displayed exceptional mechanical qualities. They also had good transparency with a visible light transmittance of 70%. With a UV light transmittance of 30%, the UV transmittance test revealed good UV-blocking characteristics. Antibacterial testing revealed that *S. aureus* and *E. coli* were inhibited^11^.

Although natural antibacterial agents are less toxic and generally safe, they have some disadvantages. The majority of them are available in trace amounts, and if used in greater quantities, they could change the flavor and odor of food. In addition, the process of extracting natural antibacterial chemicals from their sources is complicated. The amount of natural antimicrobial chemicals varies according to the original animal or plant species, which can be costly or rare. On the other hand, as food production and packaging needs increase, natural antibacterial chemicals become more scarce.

In an effort to address these drawbacks, the current study focuses on using less toxic ZnO NPs as an additional antibacterial additive with low CIN concentrations that demonstrate great efficiency in inhibiting the development of microorganisms to improve marketability. Furthermore, an examination of the combined effects of zinc oxide nanoparticles and cinnamon on the mechanical, physical, antioxidant, and antibacterial characteristics of packaging film is being conducted. Three types of gelatin based film formulation were fabricated namely: CIN@gelatin, ZnO@gelatin, and CIN/ZnO@gelatin films. CIN and gelatin, CIN@gelatin, ZnO@gelatin and CIN/ZnO@gelatin films were characterized in terms of FT-IR and XRD. The surface morphology of gelatin and CIN/ZnO@gelatin films was visualized by SEM and EDS spectra determined. The effect of CIN, ZnO NPs, and combined action of CIN and ZnO NPs on WVP, OP, mechanical, water sorption isotherm, solubility, thermal, DPPH radical scavenging capacity, and antimicrobial properties of gelatin film was investigated.

## Materials and methods

Zinc acetate dihydrate (98%) was purchased from Merck. *T*rans-cinnamaldehyde, gelatin powder from bovine skin (gel strength − 225 g Bloom, Type B) was received from Sigma Aldrich. Sodium carboxymethyl cellulose (average MW 250,000 Da, degree of substitution (0.9), DPPH radical (2,2-diphenyl-1-picrylhydrazyl) were purchased from Sigma-Aldrich.

### Preparation of Zinc oxide nanoparticles (ZnO NPs)

ZnO NPs were synthesized using sodium carboxymethyl cellulose (CMC) as a capping agent and zinc acetate dihydrate as a precursor. Zinc acetate dihydrate: The molar ratio of CMC was one to three. Initially, 20 ml of deionized water were used to dissolve zinc acetate dihydrate. CMC was then added to the solution, which was heated and agitated constantly for a whole day. Then, it is subsequently transferred to a ceramic crucible and dried for 30 min at 350 °C. This powder was calcined at 520 °C to produce crystalline ZnO NPs^27^.

### Fabrication of gelatin and gelatin composite films

As a control, 1 g of gelatin film was dissolved while being swirled in 100 mL of hot, deionized water. Next, 0.1 g of plasticizer glycerol was applied. Cinnamaldehyde-modified gelatin film (CIN@gelatin) was completed in the following ways by employing the direct emulsification technique: Tween 80 (2 wt%) was mixed with, 1% gelatin solution, and constant stirring. To establish a homogenous solution, cinnamonaldehyde (5.8%) based on the total weight of the film formulation (equal to 0.18 g) was then added to the gelatin solution with 0.1 g glycerol and stirred constantly for 2 h.

The techniques below were used to prepare the gelatin film that has been treated with cinnamaldehyde/ZnO NPs (CIN/ZnO@gelatin). After one hour of sonication, 10 mg of ZnO NPs in 50 mL of deionized water were added to a solution of 1 g of gelatin in 50 mL of deionized water, which was continuously stirred for two hours. Since the aqueous phase of the cinnamaldehyde emulsion is the gelatin/ZnO NPs solution, the CIN/ZnO@gelatin film process was repeated. Subsequently, the four film-forming solutions that were previously made were equally placed onto a Teflon film plate with a diameter of 12 cm, and allowed to dry at ambient temperature. For additional testing, the fully dried films were removed from the plates.

### Characterization

#### Structure confirmation

CIN, ZnO NPs, gelatin, and CIN/ZnO@gelatin films chemical compositions were verified by FTIR-Attenuated Total Reflection (ATR) unit attached to the FTIR-Vertex 70 Bruker, Germany, in the range of 4000–400 cm^−1^ at room temperature.

X-ray diffractions of ZnO NPs, CIN, gelatin, CIN/ZnO@gelatin films were investigated using a Bruker diffractometer (Bruker D 8 advance target). CuKα radiation source with secondly monochromator (λ = 1.5405 Ǻ) at 40 kV and 40 mA was used. 0.2 min^−1^ was the scanning rate for phase identification and line broadening profile analysis.

Using the Scherer equation (Eq. 1) to determine the average grain size of ZnO NPs.

1 $$D = \frac{0.89 \lambda }{{\beta {\text{Cos}} \theta }}$$

where λ is the wavelength (Cu Kα), β is the full width at the half- maximum (FWHM) of the ZnO (101) line and θ is the diffraction angle^28^.

#### Thermal analysis

Universal V4.5A TA Instruments SDT Q600 V20.9 Build 20 was utilized to determine thermogravimetric analysis under nitrogen gas. TG curve was used to provide kinetic studies based on data related to weight to obtain total activation energy using Arrhenius equation (Eq. 2).

2 $$\log k = {{\log \left( {\frac{dw}{{dt}}} \right)} \mathord{\left/ {\vphantom {{\log \left( {\frac{dw}{{dt}}} \right)} {\left( {w_{t} - w_{\infty } } \right)^{n} }}} \right. \kern-0pt} {\left( {w_{t} - w_{\infty } } \right)^{n} }} = \log A - \frac{{\Delta {\text{E}} _{{\text{a}}} }}{RT}$$

*w*_t_ and *w*_∞_ are mass at temperature t and final temperature, *A* is the Boltzmann constant, *R* is the general gas constant (8.314 Joules/deg. mole), *T* is the absolute temperature (Kelvin), and the *E*_a_ is activation energy. To determine the highest *R*^2^, lowest standard error for each *n*, and total activation energies, the least squares methodology was applied with 0.5 increments to* n* values ranging from 0.0 to 3.0.

#### Morphology

Morphology studies were conducted using Transmission Electron Microscopy (TEM) analysis of the as-prepared ZnO NPs was conducted using JEM-10OCXII TEM (Japan) at 120 kV. The freshly-prepared sample solutions were dropped on carbon coated copper grid to obtain a highly thin film. The sample was ready to be investigated after 15 min. Surface morphology was performed by SEM TE-scan (VEGA-3) attached to EDX Unit (Energy Dispersive X-ray Analyses), with accelerating voltage 30 K.V., magnification 14 × up to 1,000,000, and resolution for Gun.1n).

#### Permeability studies

The water vapor transmission rate (WVTR) was determined using a GBI W303 (B) Water Vapor Permeability Analyzer (China) using the cup method at (38 °C) and humidity (4%) according to a standard (ASTM E96). The gas transmission rate (OTR) was measured by an N530 Gas Permeability Analyzer (China), based on the standard ASTM D1434-82 (2003). WVP (g mm/m^2^ kPa^−1^ day^−1^) and OP (c.c/m^2^.day.atm) were calculated (Eqs. 3, 4).

3 $$WVP=S\times \frac{\text{WVTR}}{\Delta PW}$$

4 $${\text{OP}} = OPTR \times \frac{S}{\Delta PO}$$

*S* is the film`s thickness (mm) and $\Delta PW (5.942\text{ kPa})$ is the partial pressures of water vapor in saturated air with 100% relative humidity and 38 °C and air. $\Delta PO$ (0.02308 atm, at 25 °C), is the gas partial pressure difference^29,30^.

Solubility of films was determined by obtaining the initial dry mass (*M*_1_), film specimens (2 × 2 cm) precisely weighed at 0.0001 g (Eq. 5). The samples were dried at 70 °C for 24 h. The samples were then allowed to swell being kept in a Petri dish with 30 mL of water for 24 h at room temperature (25 ± 2 °C). To get the final dry mass (*M*_2_), the residual film specimens were finally dried once more in the oven under the same settings. Two measurements were made for every film sample, and the findings were reported as a percentage of the mean of the two findings^31^.

5 $$Solubility \left(\%\right)= \frac{ {\text{M}}_{1} - {\text{M}}_{2}}{{\text{M}}_{1}} \times 100$$

#### Water sorption isotherms

The impact of CIN and CIN/ZnO on the moisture sorption isotherms criteria of gelatin film was determined gravimetrically. The pre-weighed film samples (30 × 30 mm) have previously been dried at 105 °C for one day and then placed desiccators containing different salts to achieve different relative humidities at 30 oC. The samples were weighed repeatedly until there was no weight change. Equilibrium moisture contents (${\text{M}}_{\text{emc}})$ were calculated as g water/g dry film^32^ in Eq. 6.

6 $${\text{M}}_{\text{emc}}=\frac{Final weight-Initial weight}{Initial weight}$$

Two models, Brunauer–Emmitt–Teller (BET) Eq. (7) and Guggenheim-Anderson-de Boer (GAB) Eq. (8), were used to fit the experimental results.

7 $${\text{M}}_{\text{emc}} =\frac{{\text{M}}_{0} {\text{C a}}_{\text{w}}}{(1-{\text{a}}_{\text{w}}{\text{) (1+C a}}_{\text{w}}-{\text{ a}}_{\text{w}})}$$

8 $${\text{M}}_{\text{emc}}=\frac{{\text{M}}_{0}{{\text{CK}}\text{ a}}_{\text{w}}}{(1-K{\text{a}}_{\text{w}}{{) (1-}{\text{Ka}}}_{\text{w}}+{\text{CK a}}_{\text{w}})}$$

*M*_0_ is the maximum moisture monolayer, *a*_w_ is the water activity; *C* is Guggenheim constant, *K* is corrective parameter. The fit goodness of the experimental results was evaluated using Chi-square (χ^2^) (Eq. 9).

9 $$\text{Chi}-\text{Square statistics }({\chi }^{2})= \sum \frac{{(M}_{e}{, }_{\text{exp}}-{M}_{ e, }{,}_{cal{)}^{2} }}{ {M}_{e, }{,}_{cal}}$$

#### Mechanical properties

The ASTM D882–12 ASTM (2012) technique was followed when measuring the tensile strength and percentage elongation at break % of films using the Universal Testing Machine (Instron 34SC-5 Universal Machine, UK). The film specimens (8 × 1 cm), with a test segment that was 6 cm long and 1 cm wide. The experimental settings were 5 mm/min for the test speed and 0.005 N for the trigger force^33,34^.

#### Antioxidant capacity via DPPH radical scavenging ability

Concisely, 25 mg of the film was mixed with 5 ml of a 100 mM DPPH methanol solution. The reaction was kept under wraps for one hour. The absorbance of the reaction solution was determined at 517 nm^35^. The result was DPPH radical scavenging activity (Eq. 10).

10 $$DPPH\;scavening \;ability\% = \frac{{ C_{0} {-} C_{{\text{S}}} }}{{ C_{0} }} \times 100$$

where *C*_0_ and C_S_ are absorbance of blank and sample, respectively.

#### Antimicrobial properties

The inhibitory investigation of gelatin, ZnO@gelatin, CIN@gelatin, and CIN/ZnO@gelatin films against Gram positive bacteria (*Staphylococcus aureus* and *Micrococcus leutus*), unicellular yeast (Candida albicans), and Gram negative bacteria (*Escherichia coli* and *Helicobacter pylori*) was evaluated using the Kirby-Bauer disc diffusion process.

#### Cytotoxicity effect on human normal fibroblast cell line (BJ1).

The measurement of cell viability involved the reduction of yellow MTT (3-(4,5-dimethylthiazol-2-yl)-2,5-diphenyl tetrazolium bromide) to purple formazan via a mitochondrial-dependent way^36^. Cells were cultured in DMEM-F12 media supplemented with 1% l-glutamine at 37 °C and an antibiotic–antimycotic mixture containing 10,000U/ml potassium penicillin, 10,000 µg/ml streptomycin sulfate, and 25 µg/ml amphotericin B under 5% CO_2_. Using a water jacketed carbon dioxide incubator (Sheldon, TC2323, Cornelius, OR, USA), cells were batch grown for 10 days before being seeded at a concentration of 10 × 10^3^ cells/well in new complete growth media in 96-well microtiter plastic plates at 37 °C for 24 h under 5% CO_2_.

To get a final concentration of (100–50–25–12.5–6.25–3.125–0.78 and 1.56 ug/ml), media was aspirated, fresh medium (without serum) was added, and cells were cultured either alone (negative control) or with varied doses of sample. The medium was aspirated after 48 h of incubation, and 40ul of MTT salt (2.5 μg/ml) were added to each well. The wells were then incubated for an additional four hours at 37 °C with 5% CO_2_. 200 μl of 10% sodium dodecyl sulphate (SDS) in deionized water was added to each well and incubated overnight at 37 °C to terminate the reaction and dissolve the crystals that had formed. Under the same conditions, DOX, employed as a positive control, produces 100% lethality at 100 µg/ml^37^. The samples were dissolved in DMSO, which had a final concentration of less than 0.2% on the cells. Next, the absorbance was determined at 595 nm using a reference wavelength of 620 nm using a microplate multi-well reader (Bio-Rad Laboratories Inc., model 3350, Hercules, California, USA). The viability % was estimated by the below formula (11).

11 $$Viability \%=\frac{\text{Abs of control group-Abs of sample}}{\text{Abs } of control cells}\times 100$$

#### Statistical analysis

At least three replications of each experiment were conducted. Each experiment's means were available, and the findings were displayed as standard deviation (± SD) for each. Using the "SPSS" application, the data were statistically characterized using one-way analysis of variance (ANOVA) at a significance level of 0.05. The Duncan test was used to identify significant differences. The precision of the test results was measured using Chi-square (χ^2^) for non-linear fitting accuracy and correlation coefficient (*R*^2^). When the experimental data of the model closely matches the predicted data, χ^2^ will be minimal, indicating a strong fit. However, χ^2^ will be a huge number and no well-fitting will occur if the predicted data are not closer to the experimental data.

## Results and discussion

### FTIR spectroscopy

**Fig. 1:**
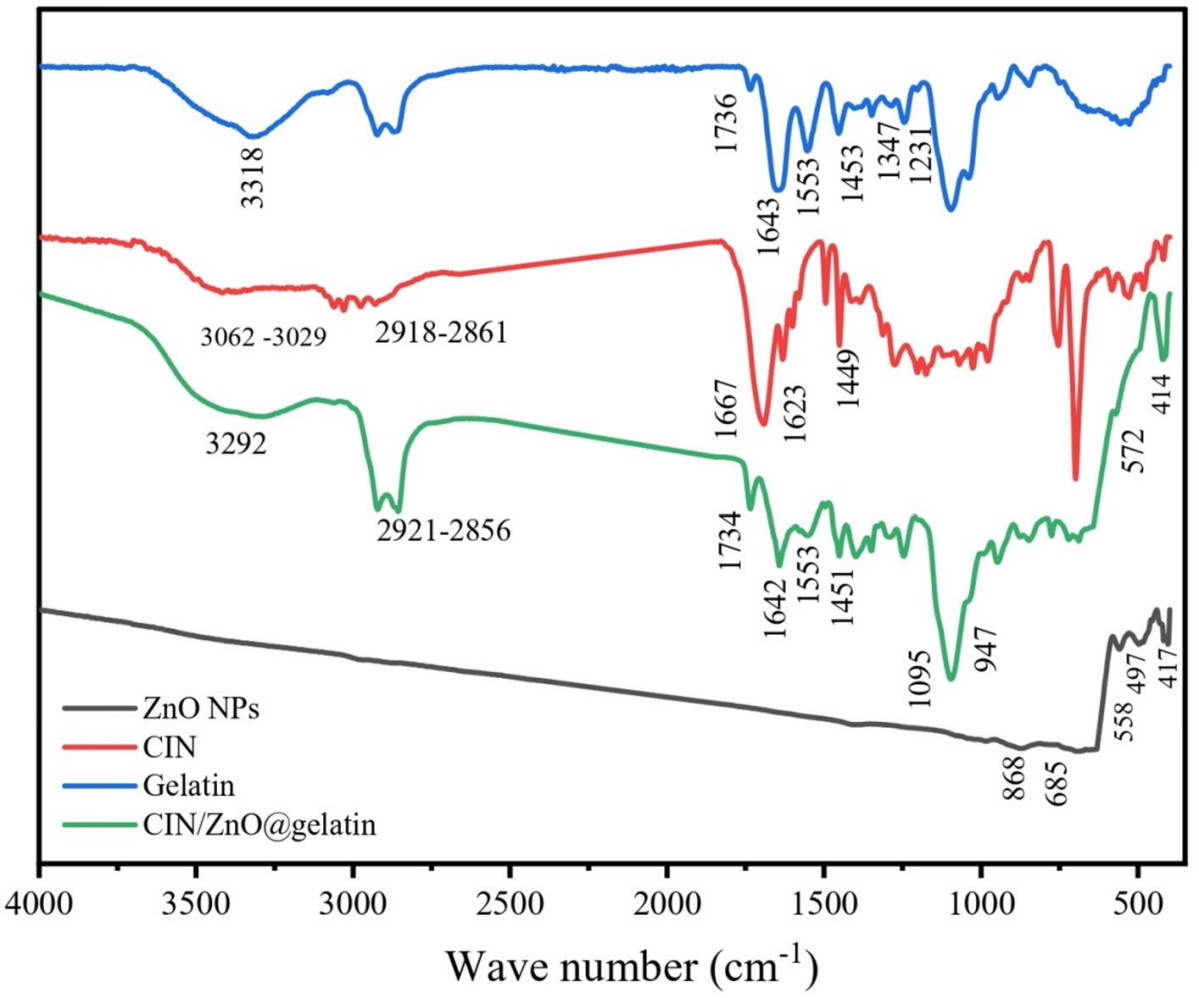
FTIR spectra of cinnamaldehyde (CIN), gelatin, and CIN/ZnO@gelatin film.

Figure 1 shows the results of FTIR spectroscopy of ZnO NPs, cinnamaldehyde, gelatin, and CIN/ZnO@gelatin films. For ZnO NPs, the absorption peaks at 868 to 417 cm^−1^ corresponded to the metal–oxygen (ZnO stretching vibrations) vibration mode^38–41^.

For CIN, the band at 1667 cm^–1^ is referred to the stretching vibration of the carbonyl group C=O of aldehyde in CIN^42,43^. The bands 1449 and 1623 cm^−1^ could be attributed to the C=C in plane vibrations of the benzene ring. A band was located at 3029–3602 cm^−1^ because of the aromatic C–H stretch. The fingerprint area of the benzene ring was observed at 1500–498 cm^–1^.

The gelatin spectrum showed an absorption band at 1643 cm^−1^ representing amide I and an absorption band at 1553 cm^−1^ attributed to amide II, originating from the C–O stretching and C–N stretching vibrations, respectively both bands were sensitive to the main chain conformation, and the band at 1231 cm^−1^ was from amide III of gelatin^44,45^. At 3318–3394 cm^−1^, amide-A showed that N–H stretching interfered with the hydrogen bonding and unbound O–H. The most informative peak for the FT-IR investigation of the secondary protein structures was amide I, which contained the C=O stretching vibration with a contribution from the C-N bond stretching vibration at 1643–1734 cm^−1^. The N–H bending vibration of amide II was observed at 1453 cm^−1^ and the C–N stretching vibration was seen at 1553 cm^−1^. Amide III denoted the plane of the C–N and N–H groups’ vibration of the bound amide or the vibration of the CH_2_ group at 1244–797 cm^−1^^46^.

The same primary peaks of the native gelatin film were visible in the FT-IR spectrum of the CIN/ZnO@gelatin film. However, hydrogen bonding and free O–H shifted from 3318 to 3292 cm^−1^ and aldehydic CO of CIN shifted from 1667 to 1642 cm^−1^ upon addition of CIN. In CIN/ZnO@gelatin film’s FT-IR spectrum, the fingerprint region of the benzene ring that lay between 1500 and 498 cm^−1^ was clearly visible. Metal–oxygen (ZnO stretching vibrations) bands at 572 cm^−1^ and 414 cm^−1^ were strappingly detected.

### XRD studies

**Fig. 2:**
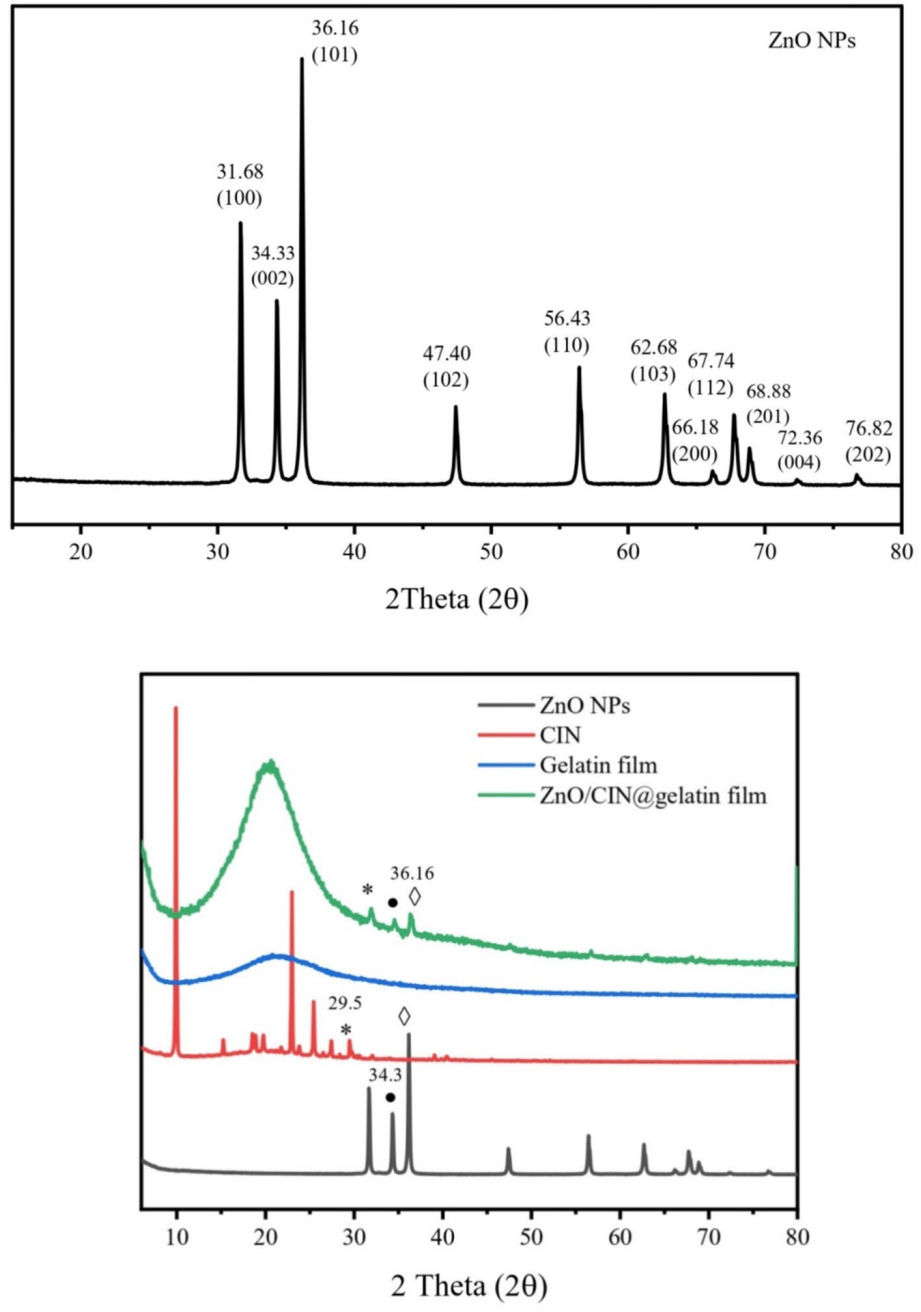
XRD patterns of ZnO NPs, CIN, gelatin, and CIN/ZnO@gelatin films.

Figure 2 verified XRD patterns of ZnO NPs, CIN, gelatin, and CIN/ZnO@gelatin films. The ZnO NPs exhibited 2θ values of at 31.68°, 34.33°, 36.16°, 47.40°, 56.43°, 62.68°, 66.18°, 67.74°, 68.88°, 72.36°, and 76.82°, respectively which corresponded to diffraction planes (100), (002), (101), (102), (110), (103), (200), (112), (201), (004), and (202), respectively. The XRD spectrum profile of ZnO verified that the synthesized ZnO NPs was the hexagonal wurtzite phase. The average grain crystallite size Scherer’s equation of ZnO NPs was formulated utilizing Scherer’s equation and has an estimated value of 1.707 nm. Mansy et al. has prepared wurtzite phase ZnO nanoparticles of average crystallite size is 20.86 nm via combustion method^47^.

The strong intensity and the narrow width of the CIN diffraction peaks, as shown in Fig. 2, suggested a high crystalline structure. Figure 2 shows gelatin’s -helix and triple-helical structure were the source of its semicrystallinity^48^. However, incorporating CIN and ZnO NPs rendered the gelatin film more crystalline. This was demonstrated by the appearance of two unique peaks specific to ZnO NPs at 34.33° and 36.16° and two distinctive diffraction peaks of CIN at 29.5°.

### Thermal studies

**Fig. 3:**
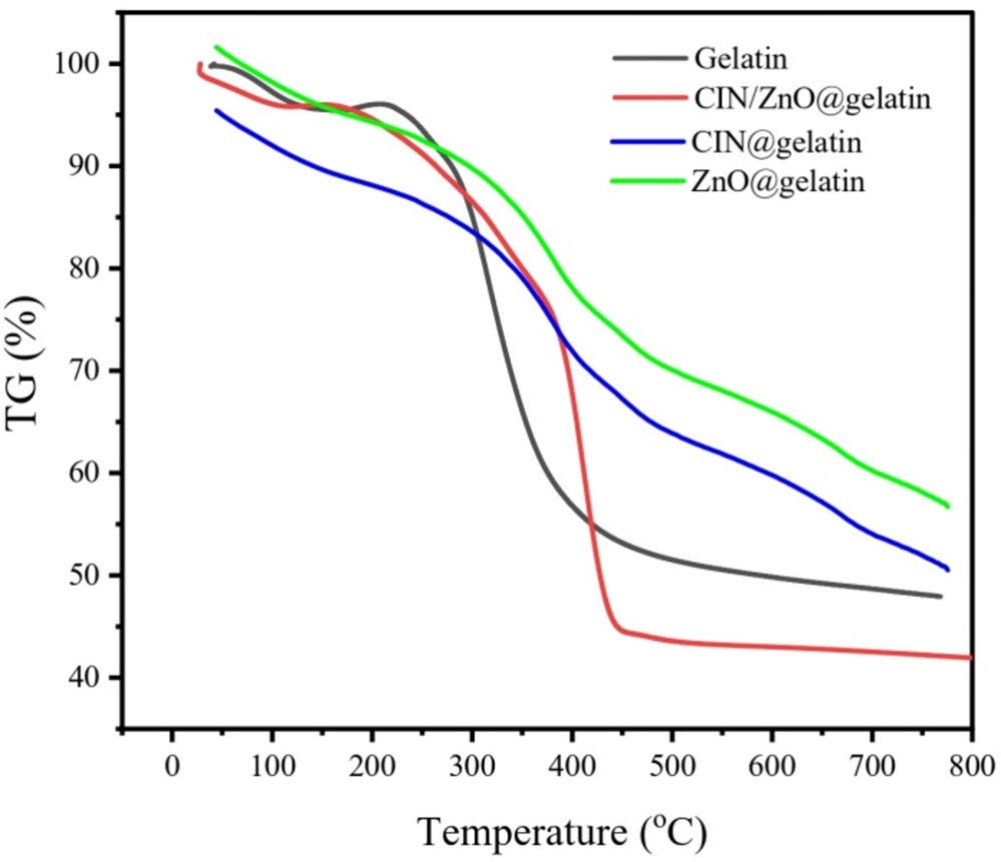
TG profile of gelatin, ZnO@gelatin, CIN@gelatin, and CIN/ZnO@gelatin films.

A typical TG thermograph of gelatin and its nanocomposite (CIN/ZnO@gelatin) is shown in Fig. 3. Weight loss percentages as a function of temperature in the range of ambient temperature to 800 °C were calculated using TG curves. Table 3 lists the phases of decomposition, decomposition temperature ranges, and maximum decomposition peak temperature for the DTG percentage mass losses. According to the thermograph, gelatin breaks down in two stages, the first of which occurred at 38.33–178.0 °C because of the moisture vaporization and a weight loss of 4.46%. The second stage of gelatin breakdown took place at 195.35–477.38 °C with a weight loss of 47.5%, which was possibly caused by ammonia loss and gelatin thermal degradation^49,50^.

ZnO@gelatin film and CIN@gelatin films degraded into two stages, the first stage was at temperature range 35.20 °C–201.11 °C and 43.91 °C–113.06 °C, respectively with maximum weight loss rate temperatures 106.20 °C and 80.41 °C, respectively and weight loss percentages 4.01% and 15.06%, respectively. The second stage begun at 206.44 °C and 173.06 °C continued to 644.18 °C and 419.95 °C with weight loss percentage 41.22% and 67.84%, respectively. The presence of ZnO enhanced thermal stability of gelatin film via increasing total activation energy by 51.08%. However, the incorporation of CIN into gelatin film decreases the total activation energy of CIN@gelatin film by 61.42%.

The CIN/ZnO@gelatin film similarly showed two phases of degradation: the first stage occurred between 40.14 and 124.08 °C and involved a mass loss of 4.17% as a result of the moisture vaporization. At 124.08–460.45 °C, the second stage had a mass loss percentage of 55.69%. The second stage’s start decomposition temperature showed a change from 195 °C to 124 °C for gelatin when CIN/ZnO was incorporated, showing that the CIN/ZnO@gelatin film was less thermally stable than gelatin. These findings matched those of the total activation energies shown in Table 1. Gelatin has nearly twice the activation energy of CIN/ZnO@gelatin. The incorporation of ZnO NPs into CIN@gelatin enhanced total activation energy of CIN/ZnO@gelatin by 11.94%.

**Table 1:** Comparative thermal degradation kinetics parameters.

Specimen	Step	Temp. set ^o^C	Max. mass loss ^o^C	Weight loss%	*R*^2^	*SE*	*n*	*E*_a_(kJ/mole)
Gelatin film	1st 2nd	38.33 -178.0 195.35–477.38	94.18 316.59	4.46 47.85	- 0.9763	- 0.199	- 3.0	- -41.01 Ʃ *E*a = -41.01
ZnO@gelatin	1st 2nd	35.30–201.11 206.44–644.18	106.20 340.69	4.01 41.22	- 0.9655	- 0.077	- 3	- -61.96 Ʃ *E*a = -61.96
CIN@gelatin	1st 2nd	43.91–113.06 173.06- 419.95	80.41 313.17	15.06 67.84	- 8688	- 0.544	- 2.5	- -15.82 Ʃ *E*a = -15.82
CIN/ZnO@gelatin	1st 2nd	40.14–124.08 124.08–460.45	81.79 412.54	4.17 55.69	- 0.8221	- 0.337	- 1.5	- -17.71 Ʃ *E*a = -17.71

### Morphology investigation

#### TEM

**Fig. 4:**
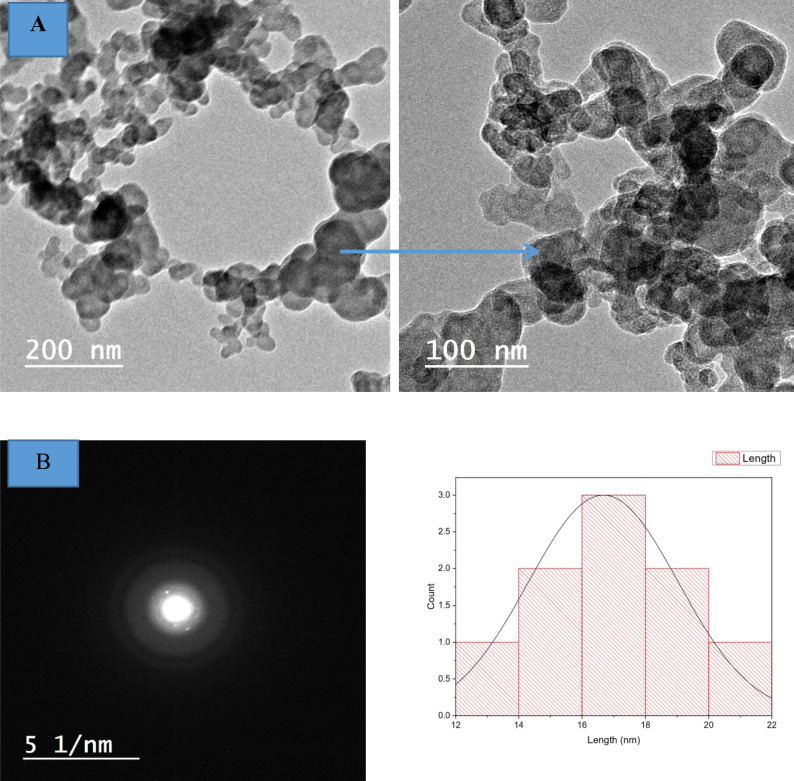
TEM images of ZnO NPs (**A**), selected electron diffraction area (**B**), particle size distribution plot.

ZnO NPs are depicted in a TEM picture in Fig. 4. The homogeneous nanocrystalline ZnO particles morphology ranged from oval to spherical in shape with an average particle size of 12–22 nm. The selected electron diffraction area of ZnO NPs implied the crystalline nature of the as-prepared ZnO NPs. The morphology of ZnO NPs as obtained by TEM pictures agrees with the findings of a study reported by Anwar et al. The fungus *Alternaria tenuissima* produces the uniform nanocrystalline ZnO particles with sphere morphologies and weak aggregation in a sustainable and environmentally friendly manner^51^. In another investigation, ZnO NPs with homogeneous and spherical morphology were produced by microwave breaking down the precursor zinc acetate, employing 1-butyl-3-methylimidazolium bis(trifluoromethylsulfonyl) imide, [bmim][NTf_2_] as a green solvent^52^.

#### SEM

**Fig. 5:**
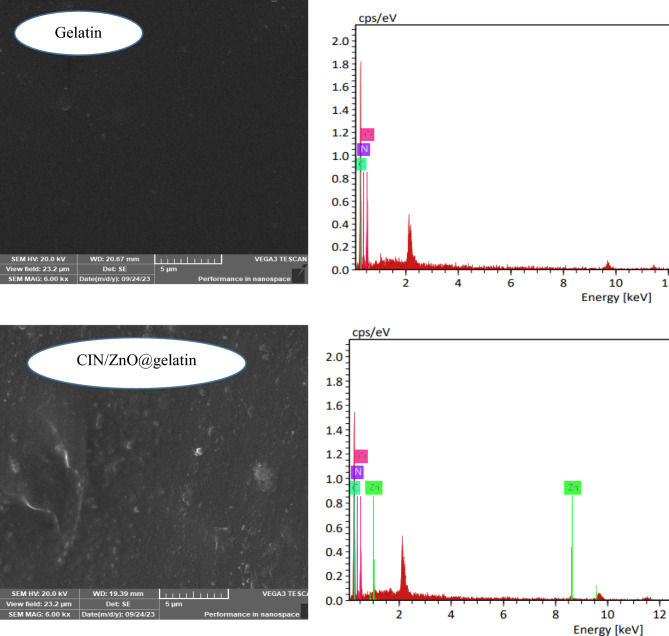

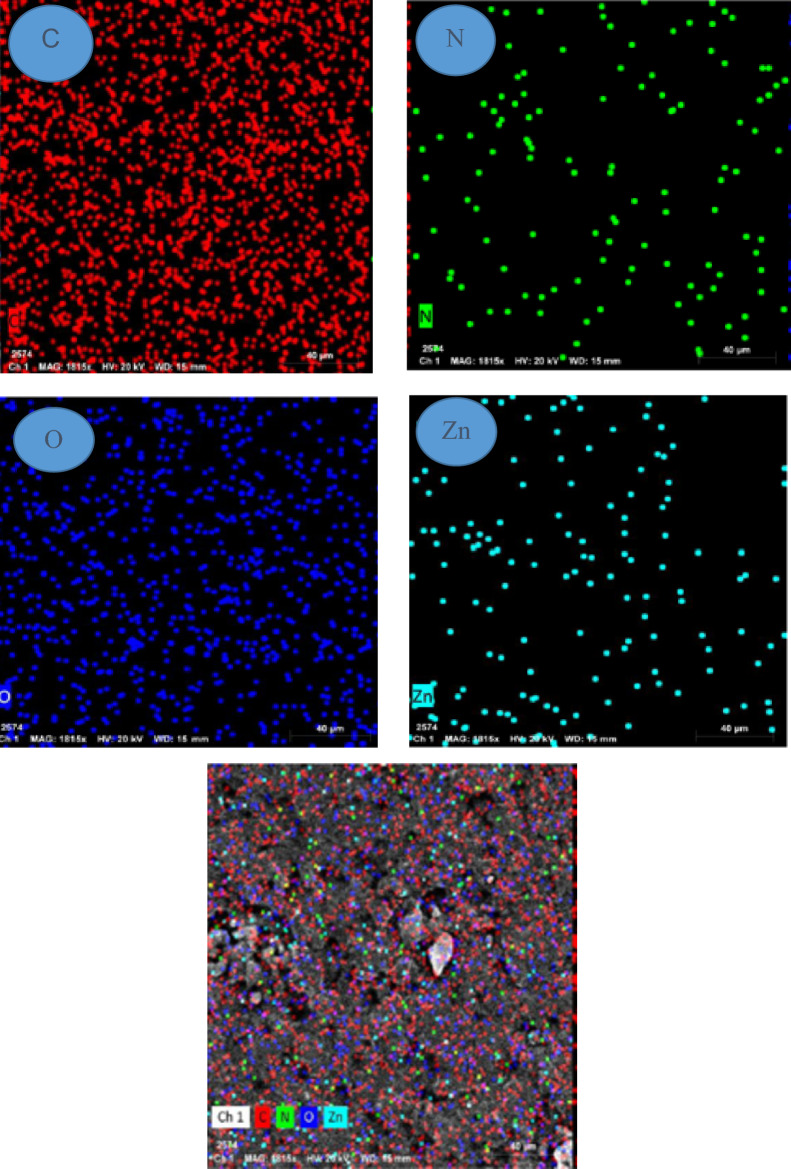
SEM images, EDX spectra, and mapping images of gelatin and ZnO/CIN@gelatin.

The morphological features of the developed composite films were analyzed by using SEM as shown in Fig. 5. The surface of the neat gelatin film was found to be smooth in nature with no air bubbles or cracking. The surface of CIN/ZnO@gelatin was observed from the SEM image to have a rough texture and an uneven surface topology because the ZnO NPs incorporated in the gelatin matrix led to deterioration in the smoothness of the surface. The fine distribution of the ZnO NPs was observed in the ZnO/CIN@gelatin composite films. The SEM analysis confirmed the continuous and the fine distribution of ZnO NPs throughout the matrix of the ZnO/CIN@gelatin composite film. Similar results were previously observed for chitosan, polyvinyl alcohol and ZnO NPs based composite films^53^. Figure 5 shows the EDS spectrum of CIN/ZnO@gelatin. Along with C, N, and O, the Zn element was observed in CIN/ZnO@gelatin when compared with the gelatin film containing the C, N, and O elements.

### WVP and OP

Table 2 presents the thickness, WVP, and OP of the gelatin, ZnO@gelatin, CIN@gelatin and CIN/ZnO@gelatin films. The native gelatin film exhibited a thickness of 0.173 ± 5.7 × 10^–3^ mm, whereas the thickness of ZnO@gelatin, CIN@gelatin, and CIN/ZnO@gelatin, was 0.19 ± 5.7 × 10^–3^, 0.193 ± 5.7 × 10^–3^, and 0.21 ± 1.0 × 10^–2^ mm, respectively. Despite the differences in the increment, the CIN@gelatin and ZnO@gelatin films showed significant difference in thickness. There was a significance difference among the gelatin film, ZnO@gelatin, and CIN/ZnO@gelatin in the thickness of the films (*p* < 0.05). This could be attributed to the increase in the solid content of the film formulation.

**Table 2:** Thickness, WVTR, OTR, WVP, OP, and solubility % of gelatin, CIN@gelatin, CIN/ZnO@gelatin.

Film parameters	Gelatin	ZnO@gelatin	CIN@gelatin	CIN/ZnO@gelatin
Thickness, mm	0.173 ± 5.7 × 10^–3 a^	0.190 ± 5.7 × 10^−3b^	0.193 ± 5.7 × 10^–3 c^	0.210 ± 1.0 × 10^−2d^
WVTR, g/m^2^ day	1583.1 ± 3.0^a^	1264.1 ± 2.0^b^	1880.1 ± 3.0^c^	1524.1 ± 2.0^d^
OTR, c.c/m^2^.day	5.25 ± 0.01^a^	0.0477 ± 0^b^	548.34 ± 1.00^c^	65.25 ± 0^d^
WVP, g.mm.m^−2^. kPa^−1^. day^1^)	46.18 ± 1.5^a^	39.00 ± 4.1^b^	61.26 ± 1.8^c^	53.86 ± 2.5^d^
OP c.c/m^2^.day.atm	0.39 × 10^–1^ ± 1.31 × 10^–3 a^	0.039 × 10^–3^ ± 1.19 × 10^–6 b^	4.593 ± 0.13^c^	0.603 ± 0.03^d^
Solubility %	41.82 ± 1.1^a^	35.86 ± 1.0^b^	56.67 ± 1.0^c^	48.08 ± 2.6^d^

^a–d^Different superscript letters in each column values represent significant difference at 5% level of probability (*P* < 0.05).

The WVTR and OTR values are included in Table 2. The insertion of ZnO NPs into gelatin film significantly decreases WVTR and OTR by 20.15% and 99.09%, respectively. However, CIN incorporation significantly increases WVTR and OTR of gelatin by 18.76% and 103.44%, respectively. The WVTR and OTR of CIN/ZnO@gelatin film significantly decreased by 18.93% and 88.10%, respectively when compared with CIN@gelatin film.

As expected, the WVP of the ZnO@gelatin film was significantly lower than that of the native gelatin film (*p* < 0.05) by 15.54%. In general, the WVP of the gelatin film tended to diminish with the incorporation of ZnO NPs, which could generally be explained by the physical crosslinking of the nanocomposite, which could diminish the diffusion of the water vapor^41^. These results were consistent with those of a study on a chitosan film previously reported by Rahman et al.^54^ and Yadav et al.^41^. In a previous study reported by Thi Minh Phuong Ngo et al. showed that the embedding of ZnO NPs in pectin /alginate film from 0 to 5 g/100 has caused a significant reduction to WVP and OP from 1.01 × 10^−14^ to 0.414 × 10^−14^ kg m/m^2^ Pa s and 270.86 × 10^−19^ to 110.79 × 10^−19^ kg m/m^2^ Pa s, respectively^55^. The ZnO@gelatin film containing 10 mg ZnO NPs was more capable of binding with water, once it presented the highest swelling index, which could explain the decrease in the WVP due to the decrease in the water vapor diffusion through the films. The ZnO NPs added to the formulation of gelatin films introduced a tortuous pathway for the water vapor molecules to pass through. According to Nielsen's simple tortuosity model, the decrease in permeability in the gelatin films with ZnO NPs could be explained as follows: each filler particle layer oriented perpendicular to the diffusion pathway, implying that for the permeability coefficient to drop, the water vapor had to take a longer diffusive path^13^. The formation of the hydrogen bonds between the gelatin and oxygen atoms in the ZnO NPs, increased the adhesion of the gelatin film matrices and decreased the diffusion of the water molecules and the reduction of the hydrophilic groups, such as NH_2_, OH, and COOH in the polymer because of the crosslinking^56^.

The CIN@gelatin film exhibited an increased WVP value higher than gelatin control film by 32.65%. The high significant WVP of CIN@gelatin film may be attributed to the inclusion of CIN, which breaks up the gelatin polymer's chain connections and creates flexible regions and an incompact structure in the film changing them to a more flexible/relaxed matrix, contributing to higher permeation of the gas^57^. Also, may be CIN acted as an internal lubricant, reducing the frictional forces between the polymer chains, and increasing the intermolecular space, thus allowing a greater mobility of the polymer chains and consequently facilitating the transport of gases^58–60^.

In contrast, the incorporation of ZnO NPs into the CIN/gelatin film significantly reduced the WVP of the CIN/ZnO@gelatin film by 12.07%. This result might be attributed to the ratio of the hydrophilic to the hydrophobic components in gelatin-based composite films as it has a direct impact on the change in WVP. The hydrophobic properties of ZnO NPs and CIN could prevent the water molecules from transferring through the film^61^.

The conclusion reported by Arfat et al. for fish skin gelatin/fish protein isolate film containing basil leaf essential oil with the incorporation of varying amounts of ZnO NPs was comparable to the findings of this investigation. The addition of ZnO NPs enhanced the film’s WVP by creating a more compact structure and lowering the rate at which the water vapor diffused through the film. When NPs were added to the film, a tortuous path was created that prevented the water vapor from passing through the film^62^. Certain nanomaterials, such titanium dioxide, could result in a polymer-based coating with a relatively low WVP^26^. Therefore, as compared to other nanoscale particles with higher hydrophilic activity, ZnO NPs might be more appropriate for use in food packaging.

The incorporation of CIN into the gelatin film significantly increased the OP of the native gelatin film from 0.39 × 10^–1^ to 4.593 c.c/m^2^ day atm (*p* < 0.05). The presence of CIN in gelatin film results in a more flexible and relaxed matrix and increased the gas’s penetration, which might be the cause of this result. By decreasing the frictional forces between the polymer chains and expanding the intermolecular space enabling the polymer chains to move more freely and thereby facilitating the transfer of gases. The incorporation of ZnO NPs led to a significant reduction of OP of the native gelatin film by 99.9% (*p* < 0.05). CIN@gelatin film had a higher OP than gelatin by 99.15%.

In contrast, the incorporation of ZnO NPs into the CIN@gelatin film significantly reduced the OP values (*p* < 0.05) by 86.86%. According to these findings, ZnO NPs might be more effective than CIN in preventing the nonpolar oxygen molecules from condensing in the film^63^.

These findings were in agreement with results of a study has been conducted by Wu et al. has investigated the functional interactions between CIN and ZnO NPs on the barrier and the characteristics of a soy protein isolate (SPI) film that was created with CIN and ZnO NPs. ZnO or CIN NPs added to the SPI matrix had varying effects on WVP and OP. ZnO NPs and CIN in an SPI-based bionanocomposite film demonstrated the best barrier aptitudes. SPI / CIN / ZnO film has an oxygen permeability of 66.1% and a water permeability of 54.8%, respectively, compared to neat SPI film^63^. Also, in another study in which the interactions between CIN/ZnO has been investigated by Xiaogang Guo et al. (2020). The carboxymethylcellulose (CMC)-based packaging film containing CIN derived from plants and ZnO NPs. The outcomes showed that adding CIN considerably enhanced the water barrier capacity performance of CMC-based films. In turn, the CMC-based composite film including CIN/ZnO NPs showed strong water and oxygen barriers^26^. The evenly dispersed ZnO NPs in the gelatin film could behave as impermeable barriers in the film, forming a tortuous route for oxygen molecules to pass through^64^. It has been shown that essential oils may not provide a particularly good gas barrier, and that the addition of essential oils typically results in an increase in the OP values of the resultant films with a high concentration^65^.

The solubility results of gelatin, ZnO@gelatin, CIN/ZnO@gelatin, and CIN/ZnO@ gelatin films is presented in Table 2. The incorporation of ZnO NPs into gelatin matrix significantly decreased the solubility of the film (*P* < 0.05) by 14.25%. This finding might be attributed to the interactions between ZnO and gelatin in the biopolymer film structure. These finding was in a consistency with a study reported by Thi Minh Phuong Ngo et al. showed that the integration of ZnO NPs into pectin/alginate film decreased the solubility of the films from 30.38 to 22.49%^55^.

The incorporation of CIN into the gelatin film significantly increased the solubility by 35.86%. This result might be related to the presence of CIN which could plasticize gelatin, changing it to a more flexible/relaxed matrix, contributing to the diffusion of free water. The plasticizers acted as an internal lubricant, reducing the frictional forces between the polymer chains, and increasing the intermolecular space, thus allowing a greater mobility of the polymer chains and consequently facilitating free water diffusion^13^. In contrast, the incorporation of ZnO NPs into the CIN@gelatin film significantly decreased the solubility of CIN/ZnO@gelatin by 15.15%. When, ZnO NPs were added to the CIN@gelatin film, a more compact structure was obtained. Additionally, ZnO NPs might cause crosslinking and reduce the solubility of the CIN/ZnO@gelatin film. Studies have reported that increasing the nanoparticle (ZnO) content of the films resulted in the formation of more hydrogen bonds in the ZnO and the matrix components. When gelatin's hydroxyl group and ZnO NPs interacted, covalent and hydrogen bonds developed, thereby increases the molecular force. Thus, free water molecules did not interact as strongly with the ZnO@gelatin film as with the gelatin films alone.

### Water sorption isotherms

**Fig. 6:**
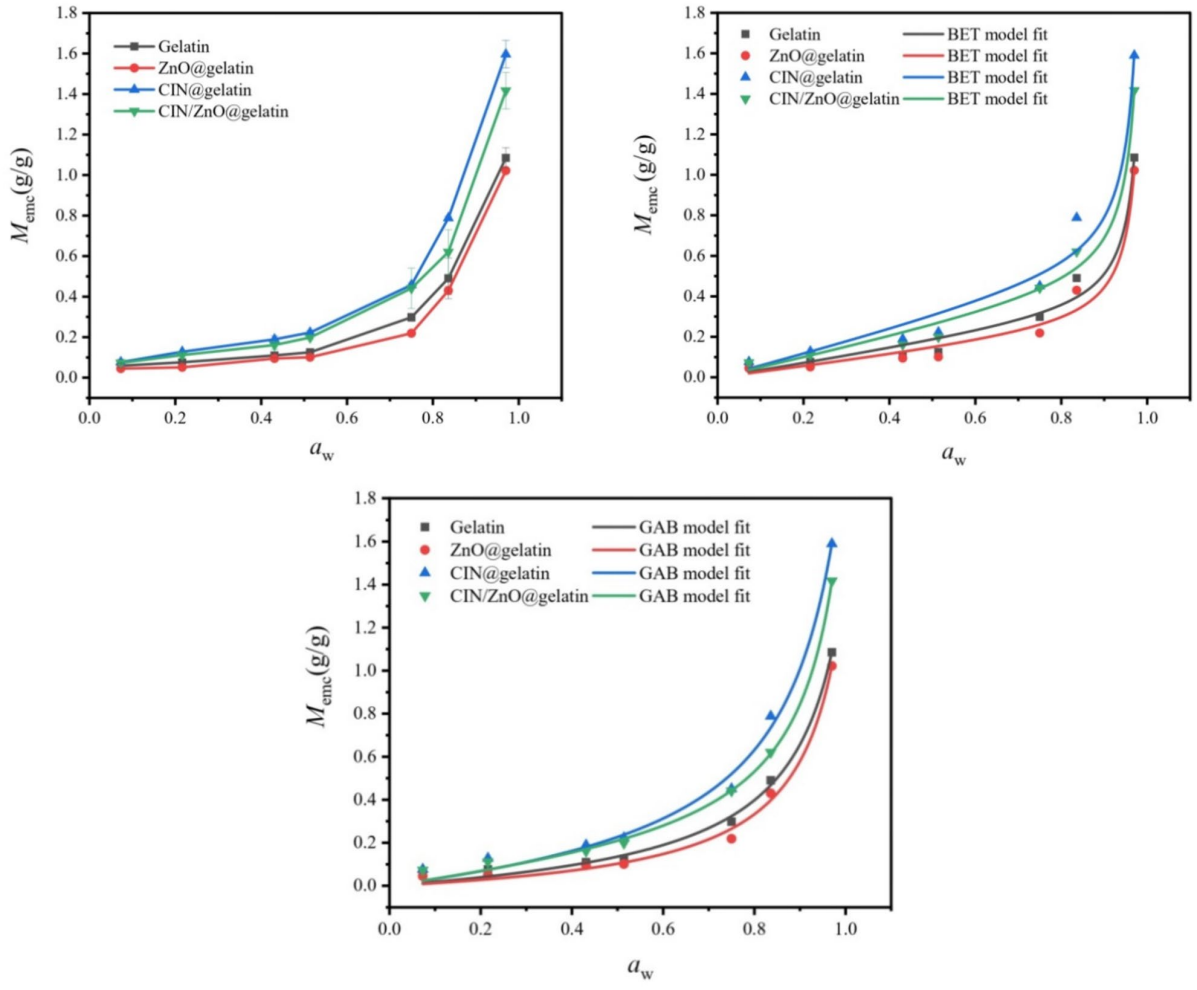
Water sorption isotherms of gelatin, ZnO@gelatin, CIN@gelatin, and CIN/ZnO@gelatin films.

Packaging films to absorb moisture is typically an important variable when assessing the durability and usability performance of these films. The equilibrium moisture contents (*M*_*emc*_) of the gelatin, ZnO@gelatin, CIN@gelatin, and CIN/ZnO@gelatin films are compared against the water activity (*a*_w_) in Fig. 6. With an increase in *a*_w_, *M*_*emc*_ effectively increased. The CIN@gelatin film had the highest equilibrium moisture content when compared with the CIN/ZnO@gelatin, gelatin, and ZnO@gelatin films, respectively. The average *M*_*emc*_ was 1.59 ± 0.06, 1.41 ± 0.09, and 1.08 ± 0.05 g/g, respectively, for the CIN@gelatin, CIN/ZnO@gelatin, and gelatin films, up to* a*_w_ = 0.97. The equilibrium moisture content of the gelatin film was influenced by the presence of CIN and ZnO. ZnO@gelatin film exhibited the lowest average *M*_emc,_ its value was 1.02 ± 0.002 g/g.

These results might be attributed to the ability of CIN at relatively high concentration (0.18 g of CIN) to plasticize polymeric films, weakening the hydrogen bonding and increasing the hygroscopicity of the films^66^. Moreover, the incorporated ZnO NPs reduced the *M*_emc_ of CIN/ZnO@gelatin by 70.32% as compared to that of the CIN@gelatin film. The incorporation of ZnO NPs resulted into a film with a more compact structure and a less hygroscopic matrix, which might be the cause of this observation^13^. This led to the decrease in the rate of water vapor diffused through the film and created a convoluted path that prevented the water vapor from passing through the film^62^.

The real data of *M*_emc_ of the CIN@gelatin, and CIN/ZnO@gelatin films in comparison with *M*_emc_ of the gelatin control were fitted using the non-linear least squares method with two moisture sorption models; the models were Guggenheim–Anderson-de Boer (GAB) and Brunauer–Emmitt–Teller (BET). The expected results were more consistent with those of the GAB model, as shown in Table 3, despite the gelatin, CIN@gelatin, and CIN/ZnO@gelatin films having greater correlation coefficients for both the BET and the GAB models. The water vapor sorption isotherm curve that was fitted with the GAB model had a higher degree of agreement with the observed data than the BET model (Fig. 6). Greater correlation coefficients were shown by the GAB model than the BET model; *R*^2^ was 0.9943, 0.9968, and 0.9922, respectively for the gelatin, CIN@gelatin, and CIN/ZnO@gelatin films, respectively (Table 3). The GAB model could be applied to determine the monolayer moisture content (*M*_0_), which was a marker of the predictable sorption characteristic. The monolayer value, which measured the number of sorbing sites, showed the maximum quantity of water that might be adsorbed in a single layer per gram of dry film (Sultan et al., 2022b). Below *M*_0_, the water vapor molecules were firmly adsorbed into the packaging material film and did not completely contribute to any deterioration processes. According to the GAB model results, it was found that ZnO nanoparticles incorporation into gelatin film significantly decreased *M*_0_ of gelatin by 48.31%. However, the incorporation of CIN into the gelatin film increased the *M*_0_ of gelatin by 64.55%. It was possible that the presence of high concentration CIN (0.18 g), which plasticizes gelatin to create a more flexible and relaxed matrix and promoted the flow and adsorption of free water, was responsible for this outcome. CIN as a plasticizer decreased the frictional forces and expanded the intermolecular space: CIN functioned as an internal lubricant, enabling the polymer chains to move more freely and thus promoting free water diffusion and adsorption, thereby causing an increase in the monolayer moisture content (*M*_0_)^13^. In contrast, the incorporation of ZnO NPs into the CIN@gelatin film reduced *M*_0_ by 35.79%. This finding could be attributed to the interactions among the plasticizer, gelatin, and ZnO NPs, which decreased the hydroxyl groups accessible for interaction with water and led to a less hygroscopic and more compact polymer film^13^. Müller et al. reported that ion–dipole interactions occur between ZnO, water, and/or plasticizer, specifically between the zinc and the hydroxyl groups of the plasticizer and water^67^.

**Table 3:** Water sorption isotherms modeling data of gelatin, ZnO@gelatin, CIN@gelatin, and CIN/ZnO@gelatin films.

BET model fitting parameters					
Sample	*M*_0_ (g/g)	*C*	*R*^2^	Chi-square (χ^2^)	
Gelatin	5.13 ± 1.8	0.068 ± 0.01	0.9787	3.51 × 10^–2^	
ZnO@gelatin	3.08 ± 1.2	0.088 ± 0.02	0.9790	3.14 × 10^–2^	
CIN@gelatin	10.10 ± 3.7	0.057 ± 0.01	0.9746	8.96 × 10^–2^	
CIN/ZnO@gelatin	8.05 ± 2.1	0.061 ± 0.01	0.9877	3.33 × 10^–2^	

GAB model fitting parameters					
Sample	*M*_0_ (g/g)	*C*	*k*	*R*^2^	Chi-square (χ^2^)
Gelatin	0.267 ± 0.30	0.696 ± 0.52	0.961 ± 0.30	0.9943	1.16 × 10^–2^
ZnO@gelatin	0.138 ± 0.21	0.901 ± 0.89	0.928 ± 0.02	0.9931	1.30 × 10^–2^
CIN@gelatin	0.932 ± 0.61	0.364 ± 0.17	0.936 ± 0.02	0.9968	1.08 × 10^–2^
CIN/ZnO@gelatin	0.535 ± 0.63	0.619 ± 0.51	0.905 ± 0.03	0.9922	3.41 × 10^–2^

Meanwhile, the GAB model correlated with the water sorption in the multi-layer BET model, and in the case of a single layer, the GAB model provided higher values of the monolayer moisture content than those produced by the BET model. Furthermore, *M*_0_ for the BET model was higher than that for the GAB; this clarified why the BET model was inappropriate.

Additionally, all graphs showed *k* as a constant 0 ≤ *k* ≤ 1 which is typically for natural films. This was according to the findings of other authors, who also stressed that the *k* parameter is effectively independent of composition^68^. The *k* constants were 0.961 ± 0.30, 0.936 ± 0.02, and 0.905 ± 0.03 for the gelatin, CIN@gelatin, and CIN/ZnO@gelatin films, respectively (Table 3).

The plot's shape was indicated by the adsorbate-adsorbent interaction suggested by the* C* constant. The magnitude of the differential between the higher layers and the monolayer was related to the *C* parameter. Every film has a *C* constant of less than 2 (*C* ≤ 2). The* C* constant values were 0.696 ± 0.52, 0.364 ± 0.17, and 0.619 ± 0.51, respectively for the gelatin, CIN@gelatin, and CIN/ZnO@gelatin films, for GAB (Table 3). This parameter decreased with the incorporation of CIN and CIN/ZnO as compared to that of the native gelatin film. Thus, the CIN@gelatin film showed a lower value than the CIN/ZnO@gelatin and gelatin films, because of the powerful plasticizing effect of CIN (Table 3). In general, the GAB model was appropriate for describing the water absorption isotherm of CIN@gelatin and CIN/ZnO@gelatin.

### Mechanical studies

**Fig. 7:**
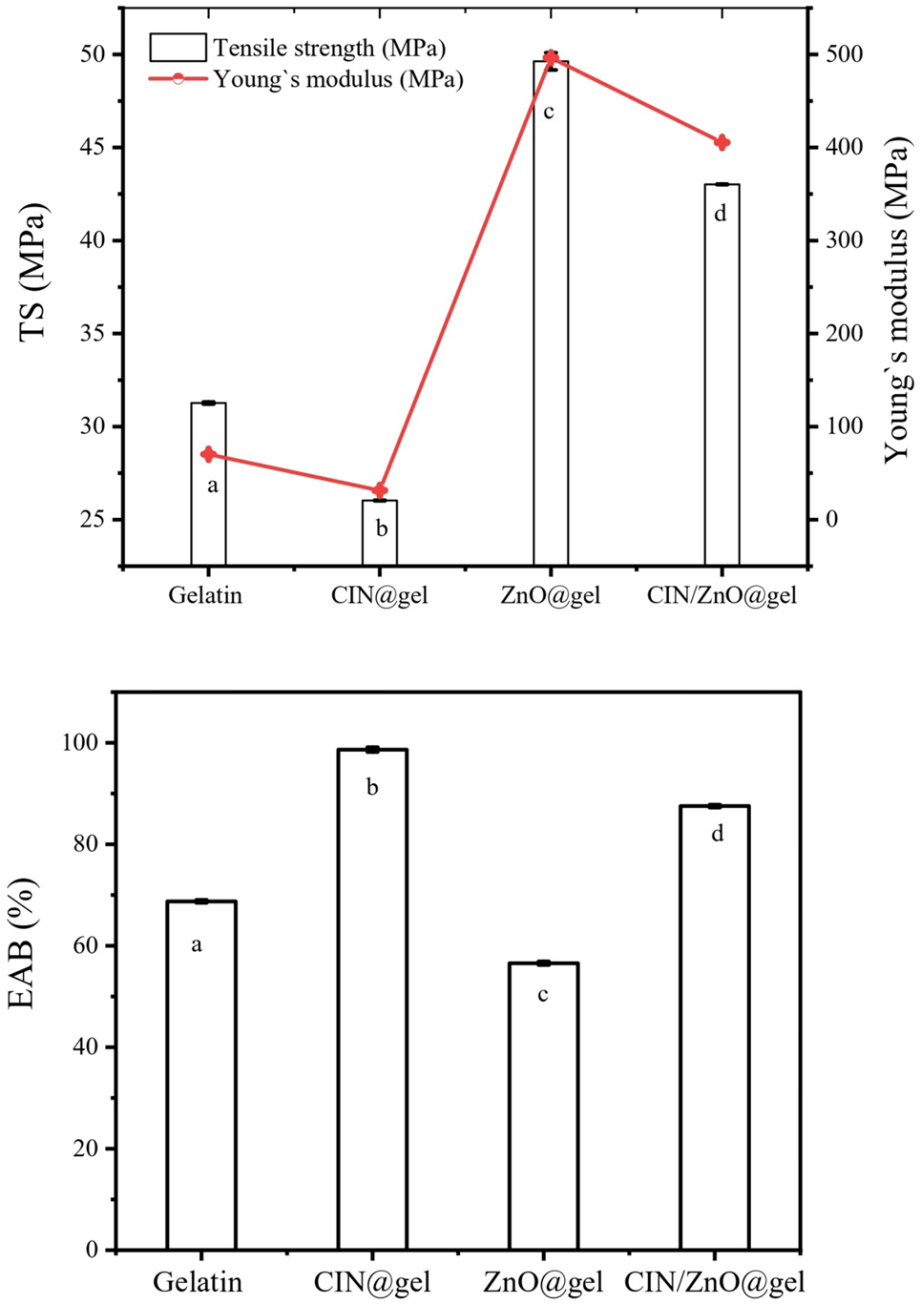
Mechanical properties of Gel, ZnO@gelatin, CIN@gelatin, and CIN/ZnO@gelatin films: a–d: Different superscript letters in each column values represent significant difference at 5% level of probability (*P* < 0.05).

The mechanical properties of the gelatin, ZnO@gelatin, CIN@gelatin, and CIN/ZnO@gelatin films are shown in Fig. 7. The reinforcement effect of ZnO NPs was significantly observed on the tensile strength (TS) of gelatin film-based formulations compared with the control film (*p* < 0.05). The incorporation of ZnO NPs into the gelatin film significantly increased the TS by 58.74%. These results were consistent with a study reported by Thi Minh Phuong Ngo et al. (2018) in which ZnO-NPs were embedded in pectin/alginate film at the concentrations of 0.5, 2.5, 5, and 25 g/100 g of blended polymer. The embedding of ZnO-NPs to blended polymer increased the tensile strength by 191.4% up to 0 to 5 g/100 g of polymer blend^55^.

Functional groups of gelatins interacted with ZnO NPs causing the formation of covalent and hydrogen bonds. Therefore, films containing ZnO NPs had a more compact film matrix structure, which resulted in the development of stronger and more rigid films^69^. However, at the same time, the incorporation of CIN into the gelatin film decreased the TS of the CIN@gelatin film by 16.74% may be due to crosslinking reason s of CIN. However, the incorporation of ZnO NPs into the CIN@gelatin film significantly decreased TS of the CIN/ZnO@gelatin film by 13.32%. Also, Young’s modulus showed the same trend, the incorporation of CIN into gelatin significantly reduced Young’s modulus of gelatin control film by 55.31% and the addition of ZnO NPs into CIN@gelatin film reduced Young’s modulus of CIN/ZnO@gelatin film by 18.33%. ZnO@gelatin film exhibited the superior Young’s modulus (496.44 ± 0.38 MPa). The addition of CIN into the gelatin film may have disrupted the protein molecules' alignment inside the film matrix, weakening protein–protein interactions and leaving the films' structure rough and incompact^57^. Also, when CIN was added, TS of gelatin films significantly decreased (*p* < 0.05). This can be explained by the presence of larger CIN droplets, which broke down intra- and intermolecular hydrogen bonds, resulting in a weaker, rougher structure and a decrease in TS^70^. In contrast, the addition of ZnO NPs can significantly improve the mechanical properties of polymer-based films as reported in many studies.

Similar outcomes have been reported by Guo et al. in a study wherein ZnO NPs and CIN were involved into a composite coating based on carboxymethylcellulose to enhance the postharvest quality of cherry tomatoes. TS of 4.91 × 107 Pa and EAB of 27.4% were displayed by the control CMC film. The EAB and TS significantly decreased to 17.4% and 3.34 × 107 Pa, respectively, upon the addition of CIN^26^. The impact of adding CIN on structural and physical characteristics of soy protein isolate-egg white composite edible film (SPI/EW/CIN) as well as their functional activity was investigated by Zhao et al. In this investigation, TS of the SPI/EW/CIN films declined progressively as the concentration of CIN increased, while EAB initially increased and then reduced as the concentration of CIN increased. The discontinuity of the film network was caused by the growth of the heterogeneous film matrix, which was encouraged by CIN^71^.

The addition of ZnO into gelatin film significantly reduced EAB% by 17.74%. Strong interactions between ZnO NPs and gelatin chains could restrict chain movements and consequently blocked its ability to flow and reduce its ductility causing decreasing elongation at break for gelatin-based formulation^63^. However, the incorporation of CIN into the gelatin film significantly enhanced EAB% by 43.48% and at the same time, the integration of ZnO into CIN@gelatin film significantly reduced by 11.27%. The addition of CIN which disrupt the chain connections of the gelatin polymer and create flexible areas in the film, may be the cause of increased levels in EAB showing that the film's ductility had significantly increased^57^. These results are in agreement with a study reported by Wu et al. have fabricated antibacterial fish gelatin film based on CIN and its sulfobutyl ether-β-cyclodextrin inclusion complex (CA/S). The addition enhanced the elongation at break and reduced tensile strength the films. EAB values of the films containing CIN and/or its inclusion complex were enhanced. The EAB % values of gelatin films containing 1% CIN and 1% CIN/sulfobutyl ether-β-cyclodextrin inclusion complex and 1% CIN were 135.03 ± 10.83% and 140.57 ± 1.80%, respectively, which were significantly improved (P ≤ 0.05). This increase of EAB was beneficial to enhance the flexibility of films. The addition of cinnamon bark essential oil reduced the TS but heightened the EAB of hagfish skin gelatin films^24^.

In a previous study, Ahmad et al. found a strong correlation between the species and concentration of plant essential oils and how they affect the mechanical properties of biocomposite films. The autours found that gelatin films with different concentrations of lemongrass oil (LO) and bergamot oil (BO) integrated have been generated and evaluated. The films' elongation at break (EAB%) and tensile strength (TS) decreased when BO and LO were added at a weight percentage of 5 to 25% (w/w) gelatin. Comparing the TS of the gelatin films with 5–25% BO and LO to the control gelatin film (33.35 ± 5.33 MPa), the values were 36.34 ± 3.1 MPa, 23.75 ± 6.85 MPa, 43.82 ± 6.56 MPa, and 21.21 ± 3.36 MPa, respectively. Comparing the EAB% of gelatin films combined with BO and LO at 5–25% (w/w gelatin) to the control (6.99 ± 3.51%), the corresponding values were 8.76 ± 3.4%, 3.06 ± 2.00%, 3.48 ± 0.92%, and 5.66 ± 2.3%^72^.

### Antioxidant activity

**Fig. 8:**
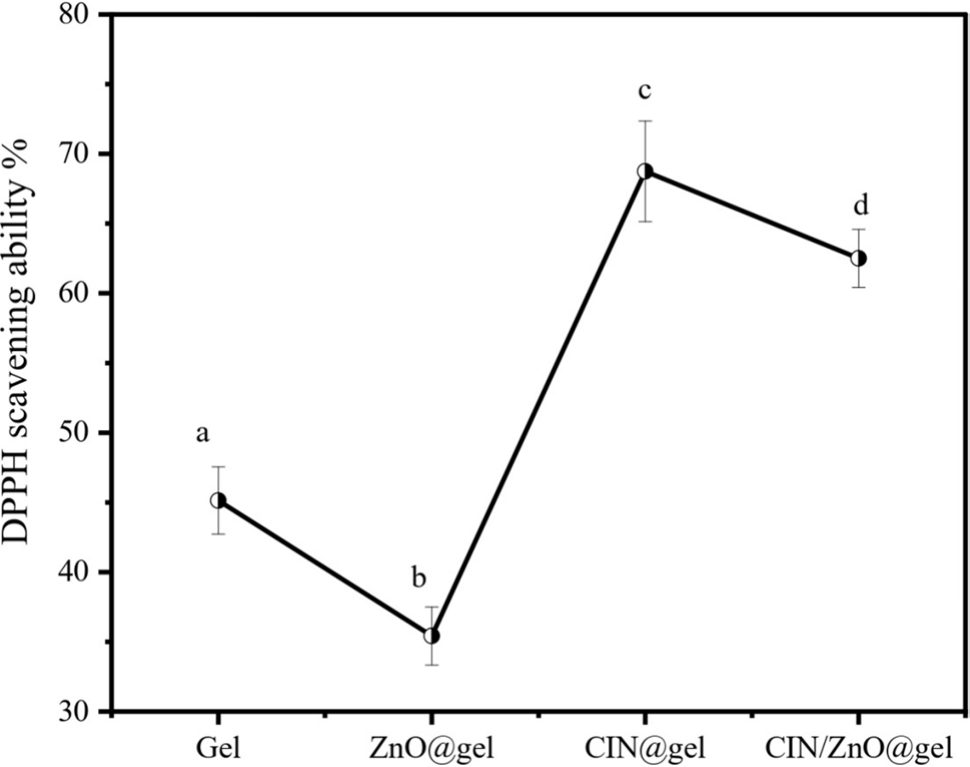
DPPH % of gelatin, ZnO@gelatin, CIN@gelatin, and CIN/ZnO@gelatin films. (**a**–**d**) Different superscript letters in each column values represent significant difference at 5% level of probability (*P* < 0.05).

One of the causes of nutrient loss and deterioration in packaged foods is the presence of free radicals. Therefore, it is crucial that food packaging materials have the ability to scavenge free radicals. The method used to survey antioxidant capacity was the 2,2-diphenyl-1-picrylhydrazyl radical scavenging test. The principle of this method was based on the reduction of DPPH in the presence of a hydrogen donating *t*rans-CIN due to the formation of diphenylpicryl-hydrozine^73,74^. The scavenging capacity of the gelatin, ZnO@gelatin, CIN@gelatin, and CIN/ZnO@gelatin films is shown in Fig. 8. Although gelatin has strong film-forming properties, it is a type of partially hydrolyzed collagen product with minimal antibacterial and antioxidant activity^75^. Some of gelatin’s amino acids are known electron donors. Consequently, a gelatin film has minimal antioxidant activity^76^. Previous studies have found that ZnO NPs can be well incorporated into a chitosan film matrix by hydrogen bonding owing to the presence of the –NH_2_ and –OH groups in chitosan. This leads to reducing amino and hydroxyl electron donors and consequently reducing the antioxidant activity^77,78^. The antioxidant activity of the gelatin film is highly affected by incorporating ZnO NPs. However, there have already been reports of small variations in the antioxidant activity of gelatin-based films containing inorganic metal oxide nanoparticles^76^. In the present study, the antioxidant capacity of gelatin film significantly reduced by 21.53% when ZnO NPs was added (*p* < 0.05). However, CIN incorporated into the gelatin film significantly enhanced the antioxidant activity of the CIN@gelatin film by 52.50%. The influence of CIN inclusion on the functional activity of soy protein isolate-egg white films (SPI/EW/CIN) has been studied by Xiaotong Zhao et al. (2021). The SPI/EW/CIN 10% film demonstrated much higher DPPH and ABTS radical scavenging activities than the SPI/EW film, reaching up to 79.37% and 66.17%, respectively. These values were nearly eight and five times higher of SPI/EW films. According to these findings, CIN exhibited strong antioxidant activity and upregulated the antioxidant enzyme heme oxygenase 1 to prevent the generation of reactive oxygen species and demonstrated that the addition of CIN significantly enhanced the antioxidant qualities of the SPI/EW films^71^.

However, the incorporation of ZnO NPs into the CIN@gelatin film caused a significant decrease in CIN/ZnO@gelatin by 9.09% (*p* < 0.05). The films enriched with CIN had a better antioxidant capacity percentage than gelatin films enriched with CIN/ZnO, and the gelatin film alone respectively. In general, the high concentration of phenolic compounds found in essential oils indicates their potent antioxidant properties. The potential cause of CIN's antioxidant impact could be its constituents’ aromatic nucleus, which have polar functional groups and can either donate or take electrons to increase the antioxidant activity of films. This could be attributed to the presence of CIN's functional groups. CIN molecule's -CHO and C=C were readily oxidized, giving the films their antioxidant ability^79^. The antioxidant capacity of gelatin films enriched with CIN/ZnO was lower than that of the CIN@gelatin film, which could be attributed to the interaction of the metal—oxide nanoparticles with CIN. The potential antioxidant activity of several nanoparticles (TiO_2_, CaCO_3_, and Al_2_O_3_) was investigated by López-Cano et al. (2023), taking into account both their pure form and modifications with cinnamon essential oil (CEO). Al_2_O_3_ loaded with CEO showed a high inhibition value of 55%, followed by TiO_2_ loaded with CEO at 28% and CaCO_3_ loaded with CEO at 35%. An 80% inhibition of the DPPH radical was shown by pure CEO^80^, suggesting that the Al_2_O_3_ nanoparticles were responsible for approximately 30% of this inhibition because of their strong association. The pure nanoparticles, in contrast, showed relatively small inhibition percentages^81^.

### Antimicrobial investigation

**Fig. 9:**
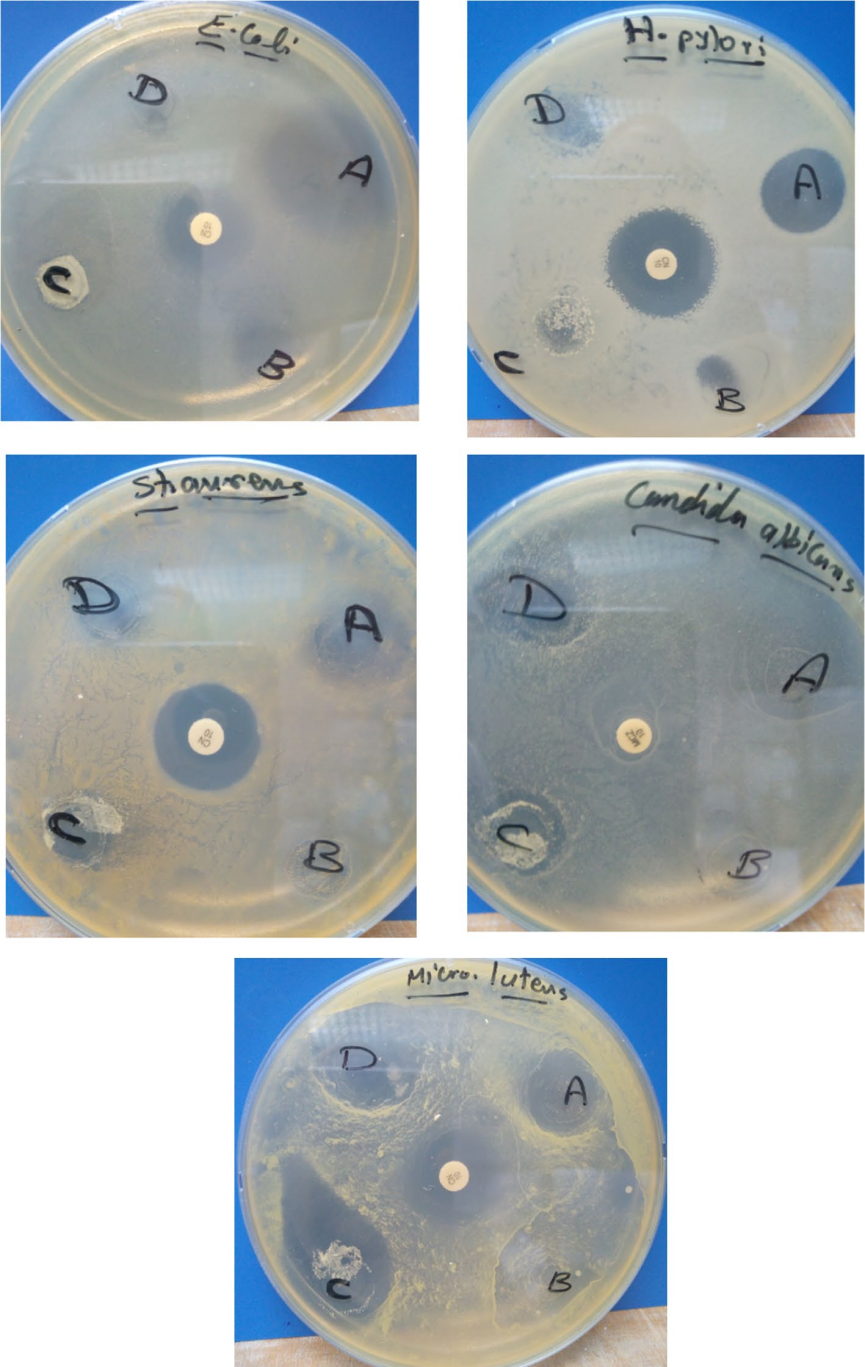
Inhibition zones generated by gelatin (**B**), ZnO@gelatin (**A**), CIN@gelatin (**C**), and CIN/ZnO@gelatin films (**D**).

Antimicrobial attributes of gelatin-based films were examined versus Gram-positive, Gram-negative bacteria, and yeast. Inhibition zone images generated by gelatin (B), ZnO@gelatin (A), CIN@gelatin (C), and CIN/ZnO@gelatin films (D) are shown in Fig. 9. The values of inhibition zones are shown in Table 4. The results of the inhibition zone revealed, in general, gelatin incorporated with CIN and ZnO NPs caused significant antibacterial and antifungal activity with varied antibacterial activities depending on the species of bacteria (*P* < 0.05). *Micrococcus leutus,* and *Helicobacter pylori* and *candida albicans* were the most sensitive microorganisms to the CIN/ZnO@gelatin film. The inhibition zone created by the CIN/ZnO@gelatin film versus *Micrococcus leutus* was 25.0 mm, which was comparable to the inhibition zone created by antibacterial gentamicin (23.33 ± 0.57 mm), and the inhibition zone created by the CIN/ZnO@gelatin film versus *Helicobacter pylori* was 15.01 ± 1.0 mm which was higher than the zone created by gentamicin (15.1 ± 1 mm). The CIN/ZnO@gelatin film created an inhibition zone against *Candida albicans* (19.0 ± 1.0 mm) was higher than zone created by miconazole (15.66 ± 0.57 mm). ZnO NPs exhibited the strongest interaction with CIN, increasing their antimicrobial capacity and their transfer of antimicrobial properties, particularly against Gram-positive bacteria and yeast. The impact of CIN/ZnO on the mechanical, physical, and antifungal aspects of a multifunctional packaging material based on soy protein isolate (SPI) has been investigated by Wu et al. The antifungal activity of the composite film containing two additional chemicals was found to be 1.56 and 1.24 times stronger than that of the SPI/ZnO and SPI/CIN films, respectively. According to these studies, the developed SPI/CIN/ZnO film has the potential to be a perfect packaging matrix for food preservation^63^. Guo et al. have studied the interactions of CIN/ZnO and their effect on the physico-mechanical and barrier assets and antifungal activities of carboxymethylcellulose (CMC)-based film packaging enriched with CIN/ZnO. The results showed that CMC-based composite film incorporating with CIN/ZnO in turn, had synergistic antifungal activity against *Aspergillus niger*^26^. According to earlier research, ZnO NPs and CIN can both have antibacterial and antifungal effects by modifying the metabolism of the reactive oxygen species^63,82^. Additionally, in light of CIN's volatility, the CIN/ZnO@gelatin composite film should be kept at low temperature and sealed to extend its usability. Consequently, the gelatin -based film that contained ZnO NPs and CIN might exhibit superior antimicrobial activity and a broad range of potential applications as active packaging in the food sector.

**Table 4:** Inhibition zones (mm) findings caused by gelatin and CIN/ZnO@gelatin films.

Tested strains	Gelatin	ZnO@gelatin	CIN@gelatin	CIN/ZnO@gelatin	Gentamicin	Micozanole
*Staphylococcus aureus*	11.0 ± 1.0^a^	18 ± 1^b^	15.66 ± 0.57^c^	17.0 ± 1.0^bcd^	19.33 ± 1.1^be^	–
*Micrococcus leutus*	15.0 ± 1.0^a^	19 ± 1^b^	22.0 ± 1.0^c^	25.0 ± 1.52^d^	23.33 ± 0.57^cde^	–
*Helicobacter pylori*	10.0 ± 1.0^a^	15 ± 1^b^	12.0 ± 1^c^	15.0 ± 1.0^bd^	19.33 ± 0.57^e^	–
*Escherichia coli*	10.0 ± 1^a^	26 ± 1^b^	15.33 ± 1.1^c^	12.0 ± 1.0^d^	15.00 ± 1^ce^	–
*Candida albicans*	12.0 ± 1.0^a^	22.0 ± 1^b^	15.66 ± 1.1^c^	19.0 ± 1.0^d^	–	15.66 ± 0.57^ce^

Positive controls: Gentamicin 30 µg (antibacterial) and Micozanole 1 mg/ml (antifungal). a–e: different superscript letters in the same raw means significance difference (*P* < 0.05).

### Cytotoxicity

**Fig. 10:**
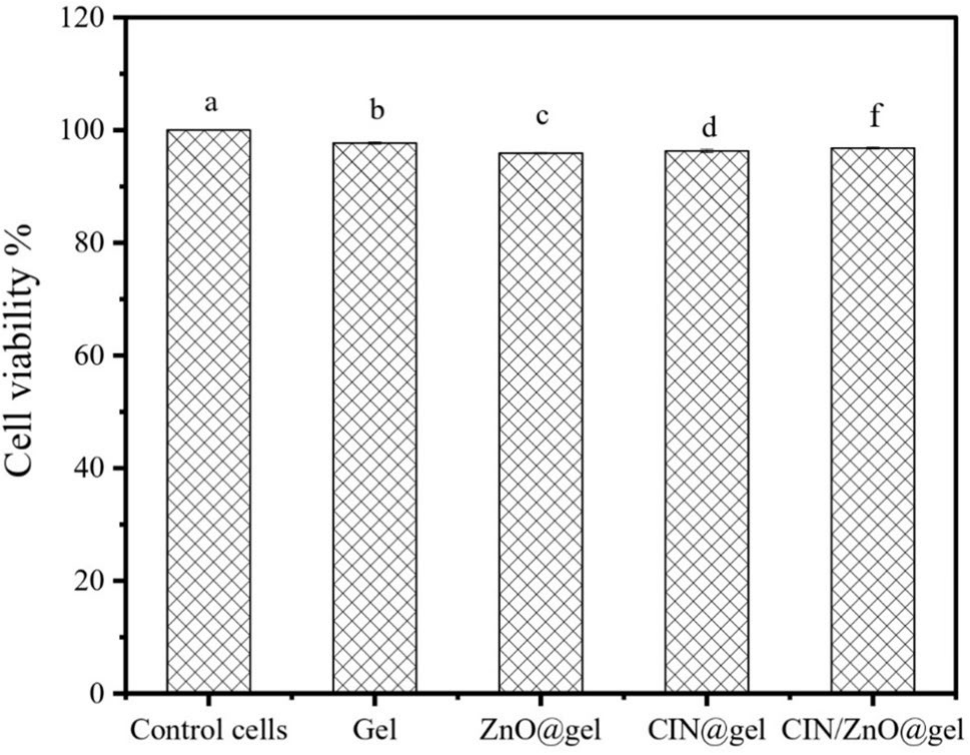
Effect of gelatin, ZnO@gelatin, CIN@gelatin, and CIN/ZnO@gelatin film samples on viability percentages of human normal fibroblast cells. The relative cell viability data for the control is the reference (100%). The mean ± SD of three independent experiments is used to express the data. Different superscript letters in each column values represent significant difference at 5% level of probability (*P* < 0.05).

The findings of cytotoxic studies carried out on normal human fibroblast cells are outlined in this section and are illustrated in Fig. 10. Comparing gelatin, ZnO@gelatin, CIN@gelatin, and CIN/ZnO@gelatin film samples to their corresponding control cells, significant changes in cell viability percentage were observed (*P* < 0.05). Gelatin, ZnO@gelatin, CIN@gelatin, and CIN/ZnO@gelatin film samples have cell viability values of 97.7 ± 0.2%, 95.9 ± 0.1%, 96.3 ± 0.3%, and 96.8 ± 0.1%, respectively. In that order when comparing ZnO@gelatin and CIN@gelatin films alone, the CIN/ZnO@gelatin film shows the maximal percentage of cell survival. As a result, it proves that the films' constituent components are biocompatible. It has been demonstrated that CIN and ZnO are potent antimicrobial and antioxidants at concentrations are not toxic to normal cells and are biocompatible, expanding the range of applications for which they may possibly be used in the development of active food packaging materials.

## Conclusion

CIN was integrated into gelatin with low toxic ZnO NPs to investigate their combination effect and interactions. ZnO NPs addition to CIN@gelatin film resulted in a considerable reduction in the film's oxygen permeability (OP) and water vapor permeability (WVP) by 12.07% and 86.86%, respectively. ZnO NPs mixing into CIN@gelatin film dramatically reduced tensile strength by 13.32%, increased elongation at break by 11.27%, and decreased Young's modulus by 18.33%. ZnO NPs integration increased the overall activation energy of CIN@gelatin by 11.94%. The Guggenheim-Anderson-de Boer (GAB) model perfectly described the water adsorption isotherms of CIN/ZnO@gelatin. The CIN@gelatin film's antioxidant activity was significantly reduced by 9.09% upon the addition of ZnO NPs. The organisms that were most vulnerable to the CIN/ZnO@gelatin film were *Micrococcus leutus*, *Helicobacter pylori*, and *Candida albican*s. The cell viability of ZnO/CIN@gelatin was 96.8 ± 0.1% revealing the biocompatibility as a food packaging film candidate.

## Data Availability

Data is provided within the manuscript.
